# Inferior vena cava injury after blunt trauma: Case report

**DOI:** 10.1016/j.ijscr.2021.105791

**Published:** 2021-03-19

**Authors:** Mohammed Aabdi, Rachid Jabi, Yassine Mellagui, Houssam Bkiyar, Mohammed Bouzinae, Brahim Housni

**Affiliations:** a Anesthesiology and Intensive Care Unit Department, MOHAMMED VI University Hospital Center, Oujda, Morocco; b General Surgery Department, Mohammed VI University Hospital Center, Mohammed I University, Morocco

**Keywords:** Blunt injury, Inferior vena cava, Trauma, Hematoma

## Abstract

**Introduction:**

inferior vena cava IVC injury is rare with lethal outcomes, the clinical signs depends on the location and associated injuries, andt he treatment might be endovascular, surgical.

**Clinical case:**

A 25 years with no medical history was admitted to the emergency department after a car accident. After intubation and hemodynamic stabilization, the computerized tomography CT scan showed hepatic laceration with a rupture of the IVC in the retro-hepatic portion, he was admitted to the operation room for damage control laparotomy; the patient died 12 h after the operation despite appropriate management.

**Conclusion:**

IVC are rare and lethal, the CT scan remains the gold standard and the evolution of endovascular techniques decreased the mortality rate.

## Highlights

• Inferior vena cava injury is a rare injury with high rate mortality.
• Few clinical cases have described the clinical findings and radiologic appearance of this kind of injury.
• We describe a rare clinical case of inferior vena cava hematoma.
• The management depends on the hemodynamic stability of the patient and the level of injury, it might be surgical, endoscopic.

## 1 Introduction

Traumatic injuries of the inferior vena cava are rare with high mortality rate and poor outcomes, occurring after penetrating or blunt trauma [[1], [2], [3], [4]]. Multiple options has been described to manage this type of injuries including endovascular repair, surgical management or non-operative management [[5], [6], [7], [8]].

In this paper, we will report the clinical case of a 25 years old man admitted to the emergency department ED after a high-speed car accident, with IVC injury and hepatic laceration.

We will represent the clinical manifestations, evaluation and management of this type of injuries.

## 2 Case report

A 25-years-old man with no medical history was admitted to the ED after a high-speed car vehicle accident.

At his admission, the vital signs were as follow: Glasgow coma score 12/15, no signs of localization, tachycardia with pulse of 126 beats/min, and hypotensive with blood pressure 75/45mmhg with no signs of external bleeding or medullar trauma and distended abdomen.

The initial complete blood count and arterial blood gas were as followed: hemoglobin 8 g/dl, hematocrit 25%, thrombopenia with platelet amount of 70,000/mm3, fibrinogen at 1.5 g/l, and prothrombin ratio 55% with severe lactate acidemia: pH of 7.07 and lactate 9 mmol/l.

Intravenous perfusion of 1 L of saline serum was initiated with norepinephrine perfusion, and the patient was put on mechanical ventilation.

Massif transfusion protocol MTP was lunched with transfusion of four fresh frozen plasma, four packed red blood cells, and four platelets units.

**Fig. 1:**
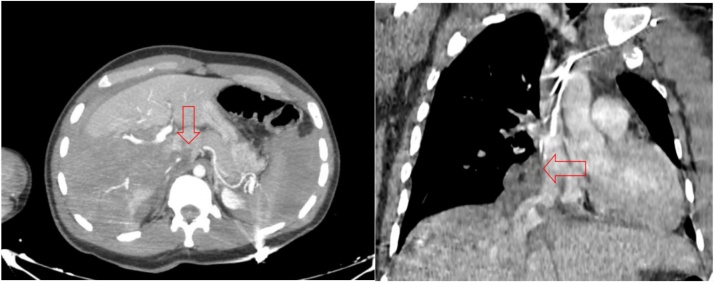
Abdomen CT scan showing rupture of the IVC (red arrow) with hepatic laceration.

**Fig. 2:**
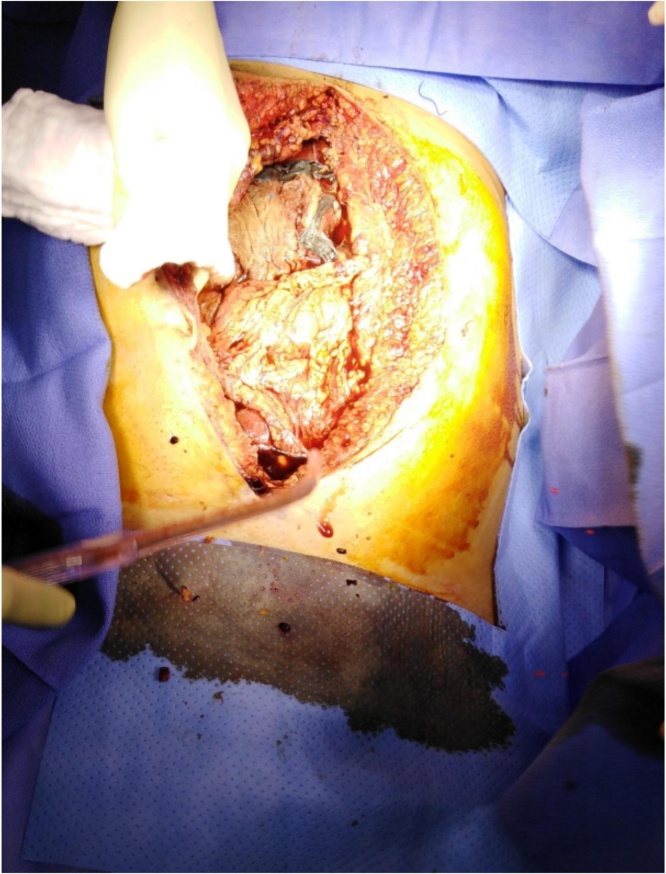
Five sponges packed around the liver.

The full body CT scan was obtained after hemodynamic stabilization, no acute injuries were notes on head and thoracic images (Fig. 1). The CT scan of the abdomen and pelvis revealed massive hemoperitoneum and multiples liver lacerations extending to the IVC (Fig. 2).

The patient was taken to the OR for damage control laparotomy. Upon opening the abdomen, he was actively bleeding with visualization of large laceration of the right liver. The decision was made to leave five sponges packed around the liver and leave the abdomen open for a second look surgery.

The patient turned to the intensive care unit, he kept worsening on clinical and biological levels: hypotensive despite MTP, norepinephrine infusion and continuous veno-venous hemofiltration with apparition of multi-organ failure: kidney failure, liver failure, consumptive coagulopathy and severe lactic academia (pH 6.9, lactate 12 mmol/l).

The patient died after 12 h with multi-organ failure.

This case report follows SCARE guidelines [9].

## 3 Discussion

Injuries of the IVC are rare occurring in 0.5–5% and 1% of penetrating and blunt trauma respectively with high rate mortality: 50% upon hospital arrival and up to 70% in-hospital mortality [[10], [11], [12], [13], [14], [15]]. The mean cause of death is due to uncontrollable intraoperative hemorrhage [16,17].

Penetrating IVC injuries are usually associated with organs and vessels injuries: liver, duodenum, and pancreas are most likely to be injured [13].

Anatomically, the most frequently injured segment of the IVC is the infra-renal segment (39%), followed by the retro-hepatic segment (19%), the supra-renal segment (18%), the para-renal segment (17%) and the supra-hepatic segment (7%) [14].

Anatomical location, associated injuries, physiological status, Glasgow coma scale, shock state and absence of hemodynamic response to volume substitution protocols are predictors of mortality in the IVC injuries [14,15,18,19].

CT scan of the abdomen and pelvis is the gold standard in the diagnosis of the IVC injuries and it should be performed after hemodynamic stabilization of the patient [13,20].

After initial resuscitation of the patient, the management of the IVC injuries remains a challenging issue to the medical team: surgical, endovascular or non-operative management.

The evolution of endovascular techniques like temporary balloon and resusvitative endovascular balloon aortic occlusion REBOA have decreased the morbidity and mortality in those injuries [21].

Non-operative management of retro-hepatic IVC injuries have been describes [[22], [23], [24], [25]].

Surgical management depend on the condition of the patient, level of the injury and its extent, and the expertise of the surgical, anesthetic and nursing team, caval ligation and venorrhaphy are the most adopted strategies [13].

## 4 Conclusion

Traumatic injuries of the IVC are quite rare with high morbidity and mortality rate, the CT scan is the gold standard for the diagnosis of IVC injuries and the associated lesions. The management depend on the location of the injuries and hemodynamic stability of each patient.

## Declaration of Competing Interest

The authors report no declarations of interest.

## Sources of funding

This research did not receive any specific grant from funding agencies in the public, commercial, or not-for-profit sectors.

## Ethical approval

The ethical committee approval was not required given the article type (case report).

## Consent

Written informed consent was obtained from the patient’s father for publication of this case report and accompanying images.

## Author contribution

AABDI Mohammed: study concept, Data collection; data analysis; writing review & editing.

Jabi rachid: Study conception, data analysis.

MELLAGUI Yassine: contributor.

BKIYAR Houssam: contributor.

BOUZINAE Mohammed Supervision and data validation.

HOUSNI Brahim: supervision and data validation.

## Registration of research studies

Not applicable.

## Guarantor

AABDI Mohammed.

JABI Rachid.

## Provenance and peer review

Not commissioned, externally peer-reviewed.
